# A computational framework for inferring species dynamics and interactions with applications in microbiota ecology

**DOI:** 10.1038/s41540-025-00568-0

**Published:** 2025-08-05

**Authors:** Yuanwei Xu, Georgios V. Gkoutos

**Affiliations:** 1 Department of Cancer and Genomic Sciences, College of Medicine and Health, University of Birmingham, Birmingham, UK; 2 Centre for Health Data Science, University of Birmingham, Birmingham, UK; 3 Centre for Environmental Research & Justice (CERJ), University of Birmingham, Birmingham, UK; 4 Present Address: Liangzhu Laboratory, Zhejiang University School of Medicine, Hangzhou, China

**Keywords:** Computational biology and bioinformatics, Systems biology

## Abstract

We present MBPert, a generic computational framework for inferring species interactions and predicting dynamics in time-evolving ecosystems from perturbation and time-series data. In this work, we contextualize the framework in microbial ecosystem modeling by coupling a modified generalized Lotka-Volterra formulation with machine learning optimization. Unlike traditional methods that rely on gradient matching, MBPert leverages numerical solutions of differential equations and iterative parameter estimation to robustly capture microbial dynamics. The framework is assessed within the context of two experimental scenarios: (i) paired before-and-after measurements under targeted perturbations, and (ii) longitudinal time-series data with time-dependent perturbations. Extensive simulation studies, benchmarking on standardized MTIST datasets, and application to *Clostridium difficile* infection in mice and repeated antibiotic perturbations of human gut micribiota, demonstrate that MBPert accurately recapitulates species interactions and predicts system dynamics. Our results highlight MBPert as a powerful and flexible tool for mechanistic insight into microbiota ecology, with broad potential applicability to other complex dynamical systems.

## Introduction

Many natural and engineered systems consist of interacting species whose behaviors evolve over time. In such systems, the state can be represented by a time-dependent vector of measured quantities, while the mechanisms driving species interactions remain largely unknown. One common modeling approach is to describe the system dynamics with a set of Ordinary Differential Equations (ODEs) whose unknown parameters capture the interaction effects and other key properties. External perturbations–whether they target specific species or act indirectly–provide a means to modify the system state and quantitatively assess the impact on species dynamics. By estimating these ODE parameters, one can infer potential interactions and, using the resulting model, predict how the system will evolve under new initial conditions or perturbations. A prime example of this modeling framework is the ecological system of microbiota, whose dynamics are often modeled using the generalized Lotka-Volterra (gLV) equations; here, perturbations may include bacteriotherapy, antibiotics, or dietary interventions.

Microbiome communities exist in diverse habitats and exhibit unique functions and compositions dictated by their hosts and environments. In the plant microbiome, for example, microbes colonize distinct niches within plant tissues and play essential roles in plant physiology and metabolism^1^. Compounds produced by soil microbes can influence plant signaling and trigger the production of microbe-derived compounds^2^. Such interactions are critical for plant health and productivity, as they stimulate growth, enhance stress resistance, and improve nutrient uptake and transport^3–5^. In the oceans, each milliliter of the 1.3 billion km^3^ of water contains millions of microbial cells, and marine animals form close associations with vast numbers of microorganisms^6^. The oceans rely on these organisms to drive Earth’s biogeochemical cycles, maintain oxygen production, and facilitate nutrient cycling and matter degradation^7–10^. With the looming challenges of climate change and anthropogenic impacts, the ocean microbiome may even serve as a sentinel for environmental adaptation in marine animals^11^.

Humans, too, host a wide array of microbiome communities across various body sites, with the gastrointestinal tract being the most diverse microbial habitat. Gut microbes perform critical functions ranging from digesting food to modulating the host immune system and metabolism^12–15^. Disruptions in the gut microbiota–referred to as dysbiosis–have been linked to the development and progression of numerous diseases, including diabetes^16,17^, obesity^16,18,19^, inflammatory bowel disease^20^, neurodegenerative diseases^21^, and cancer^22–25^. Recent findings indicate that the gut microbiome in colorectal cancer patients exhibits a reduced number of microbe-microbe associations, suggesting that interventions targeting single species may be insufficient to restore a healthy state^26^. Such observations underscore the importance of collective microbial interactions–which cannot be captured by studying individual species in isolation–for understanding community function^27^, assembly^5^, and stability^28^, as well as for predicting responses to perturbations such as antibiotics or dietary shifts^29–31^.

The ability to manipulate and restore microbiome communities has garnered significant interest both practically and theoretically. For instance, soil microbes can facilitate the hydrological restoration of degraded land and promote plant growth^32^, while fecal microbiota transplantation (FMT) has been successfully used to treat recalcitrant Clostridium difficile infections (CDI)^33^. In addition, graph-theoretical control frameworks have been developed to identify minimal sets of driver species capable of steering the system toward a desired state^34^. Designing personalized therapeutic interventions and bacteriotherapies remains an active area of research^35–37^. However, the complex, bidirectional interactions–often involving unknown taxa and mechanisms of cooperation and competition–pose substantial challenges for conventional modeling approaches^38,39^.

Ecological dynamical system models based on the gLV equations have long been employed to predict species dynamics and responses to new perturbation events. The parameters in these equations encode key ecological properties and can be inferred from microbiome time-series data^29–31,40^. From the estimated interaction matrix, de novo species interaction networks can be constructed. Unlike simple correlation networks, these data-driven networks are directed, signed, and weighted, potentially encoding causal mechanisms^41,42^. Moreover, analyses of stability and keystoneness enable the assessment of which species combinations are likely to coexist and the marginal importance of each species within the community^30^.

A common approach to estimate the gLV parameters is the so-called “gradient matching” algorithm^29,30^. This method discretizes the gLV equations, transforming them into a system of linear equations that can be solved using regularized linear regression techniques. However, gradient estimates can be inaccurate are prone to error, especially when microbiome data are sparsely sampled or collected at time points close to equilibrium, which can lead to significant inaccuracies in the inferred parameters^30,40^. Inspired by recent work that combines interpretable machine learning with dynamical systems to extract biological insights from drug perturbation-response data^43^, we propose a generic computational framework called MBPert. This method allows for accurate prediction of microbial dynamics and estimation of species interaction networks from both perturbation and time-series data without relying on gradient matching. MBPert is designed to be flexible in handling different types of experimental designs, specifically: (a) paired measurements across multiple targeted perturbations and (b) longitudinal sampling with potential time-dependent perturbations. MBPert thus expands the applicability of dynamical systems modeling to a broad range of experimental scenarios and application domains. MBPert has been implemented in PyTorch, leveraging its optimized machine learning framework and GPU computation. We demonstrate the framework using bacterial time series data of *Clostridium difficile* infected gnotobiotic mouse^30^, and also the human gut microbiome data of repeated courses of the antibiotic ciprofloxacin^44^.

## Results

### The general framework and contextualization in microbial dynamics modeling

**Fig. 1:**
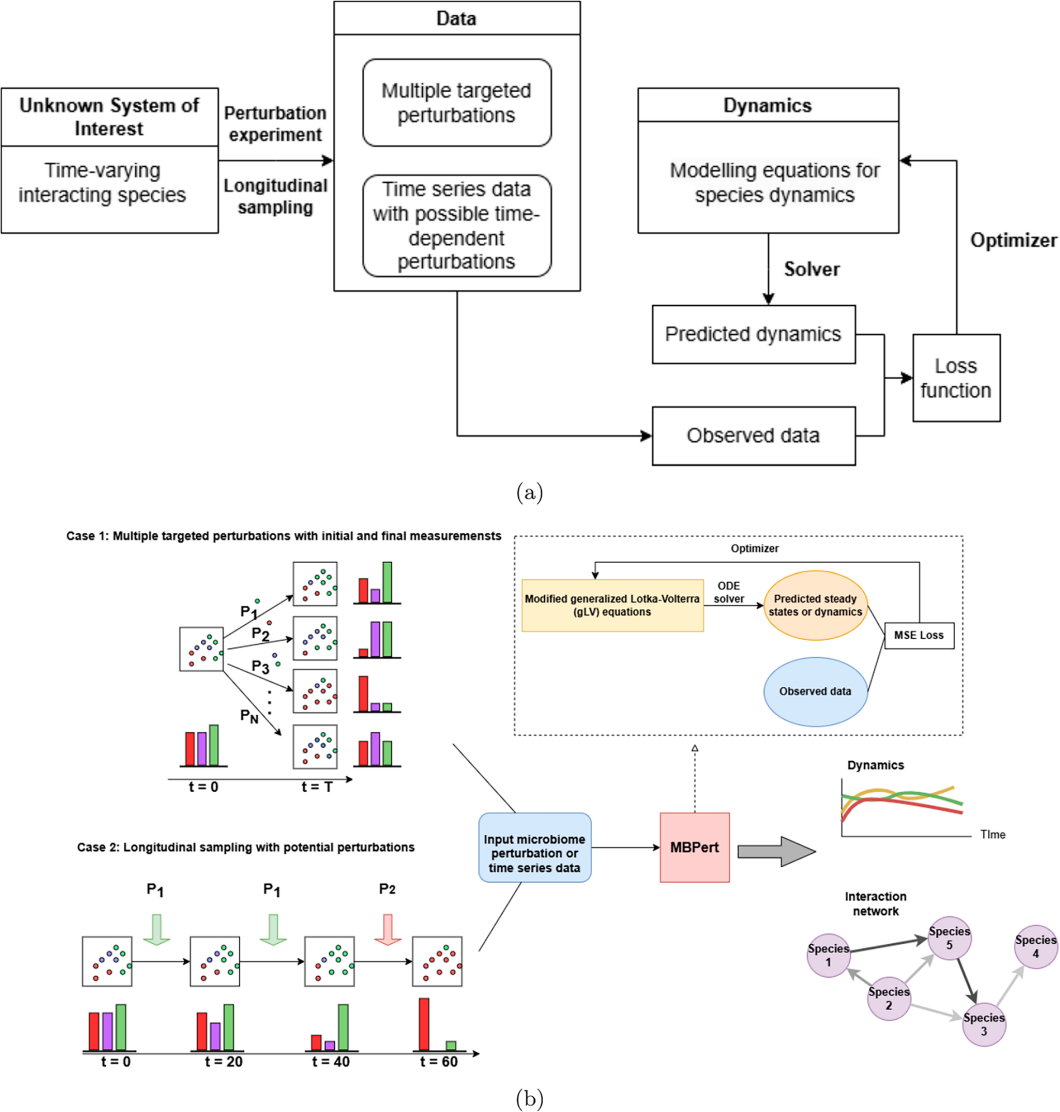
Illustration of MBPert. The general framework for species interaction inference and dynamics prediction based on different types of input data (**a**); and applying this framework in the context of microbial dynamics, where the input is microbial perturbation or time series data, the modeling equations are the gLV equations and the loss function is the mean squared error loss (**b**).

The proposed generic computational framework is depicted in Fig. 1a. We consider two types of input data. The first consists of system states profiled before and after multiple targeted perturbations. The second is time series data with possible time-dependent perturbations. The framework combines dynamical system and machine learning optimization to estimate the parameters in an iterative fashion. Given the current estimate of the parameters, the differential equations are numerically solved to produce a predicted system state, which is then compared against the observed data. The optimizer then seek to update the parameters to minimize the difference between these two quantities.

In the context of computational microbiome dynamical modeling, we used the gLV equations as the governing equations with appropriate modifications to account for perturbation events. The method, MBPert, can be applied to either targeted microbial perturbations data (Case 1), or time series data with potentially different perturbation types (Case 2) (Fig. 1b).

### Simulation study of paired measurements before and after perturbations

To test the performance of MBPert, we conducted comprehensive simulation study using synthetic data, while accounting for different scenarios with practical significance.

We considered 10 species, for which the total number of combinatorial perturbation conditions is 2^10^ − 1 (minus the case when no species are targeted). MBPert was trained using the initial and steady states across all perturbation conditions specified by a binary matrix *P* with ${P}_{i}^{\mu }=1$ if perturbation *μ* targets species *i* and ${P}_{i}^{\mu }=0$ otherwise. The following simulation setups differ in the choice of *P* and how the data was partitioned during training and validation.

First, the perturbations were randomly split. Here *P* includes all single-species perturbations and 50% of all combinatorial perturbations of up to 5 species. We compared the MBPert estimated gLV parameters with the exact values, and the predicted steady states with the exact ones under validation set perturbations.

**Fig. 2:**
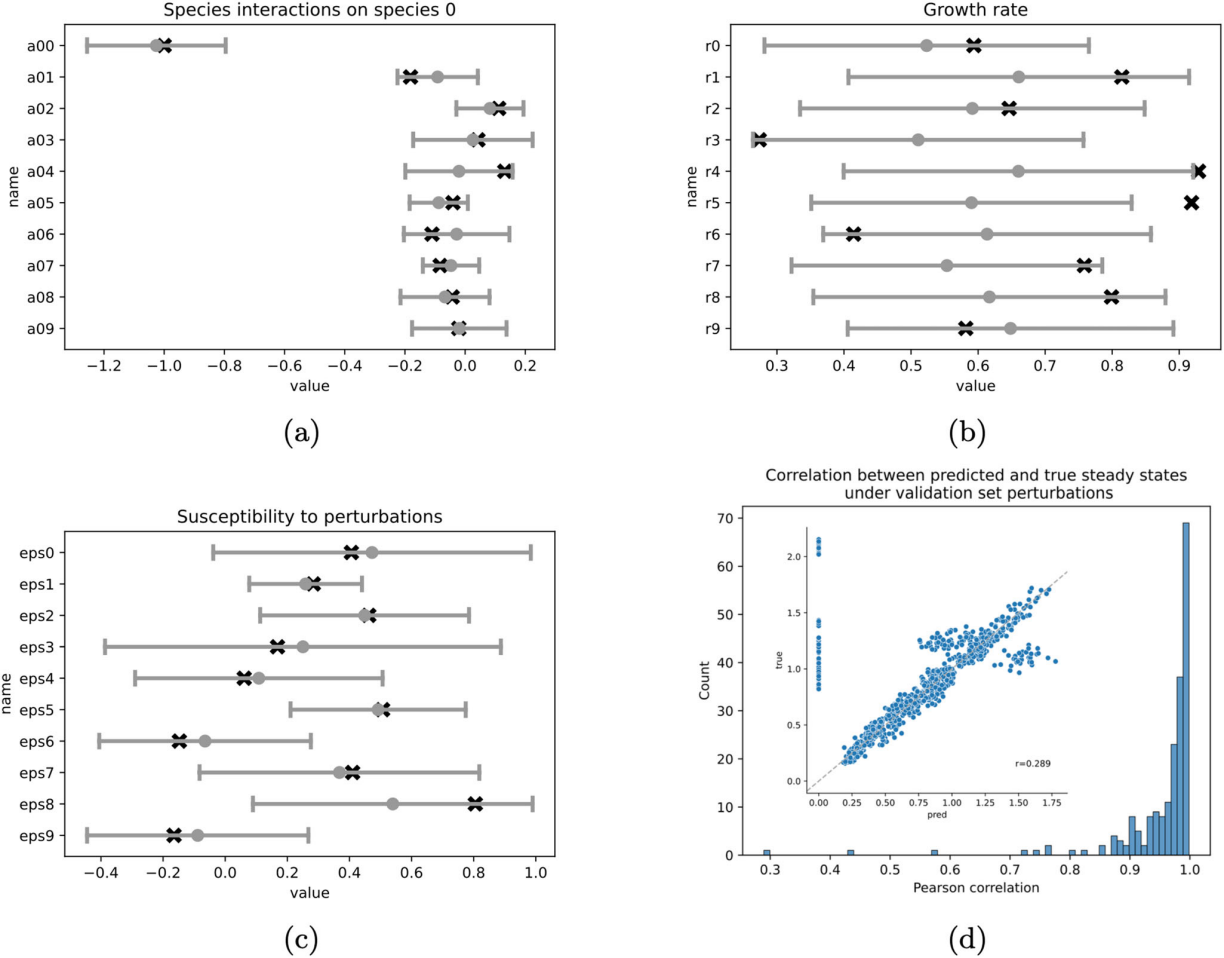
Randomly splitting perturbations in the simulation study of paired measurements across multiple targeted perturbations. 323 perturbations was randomly split into training and validation sets and MBPert was trained on the initial and steady states corresponding to training set perturbations and evaluated on validation set perturbations. This was repeated for 200 times. In (**a**–**c**), the interval represents the mean and one standard deviation of the estimate over all training rounds. The exact values are indicated by black crosses. **a** Interaction effect on species 0, corresponding to the first row of *A*. **b** Growth rate **r**. **c** Species overall susceptibility to perturbations. **d** Histogram of the Pearson correlations between predicted and true steady states under validation set perturbations. Inset shows the predicted and exact values for the lowest correlation case. The low correlation was caused by a small proportion of non-trivial steady states that were incorrectly predicted to vanish.

We found that 90 out of 100 species interactions, 8 out of 10 growth rates, and all susceptibility values fell within one standard deviation of the corresponding MBPert estimates (Fig. 2a–c, Supplementary Fig. 1). All parameter values fell within two standard deviation of the corresponding MBPert estimates. Pearson correlation between predicted and true steady states was computed for each validation set perturbations and its distribution was concentrated near one (Fig. 2d), showing good agreement between predicted and true steady states for unseen perturbations. There were some low correlations observed (minimum *r* = 0.289). However, closer examination showed that this was caused by a small proportion of non-trivial steady states that were incorrectly predicted to vanish, whereas the majority of steady states aligned with the exact values (inset of Fig. 2d). We hypothesize that this could be caused by the algorithm being stuck at a local minimum in the high-dimensional parameter space. However, most of the runs tended to converge to the global minimum, since only 3 out of 200 (1.5%) runs had correlations less than 0.7 (histogram in Fig. 2d).

Second, the perturbations were split by the number of targeted species. Suppose we only observe the system under monospecies perturbations, can we predict species dynamics under new, combinatorial perturbations? This is a much harder task than random splitting because our training set would contain very limited information: just 10 perturbations, each targeting a single species. An easier task would be to train the model on perturbations involving all single and pairwise species and test its performance on perturbations targeting more than two species.

**Fig. 3:**
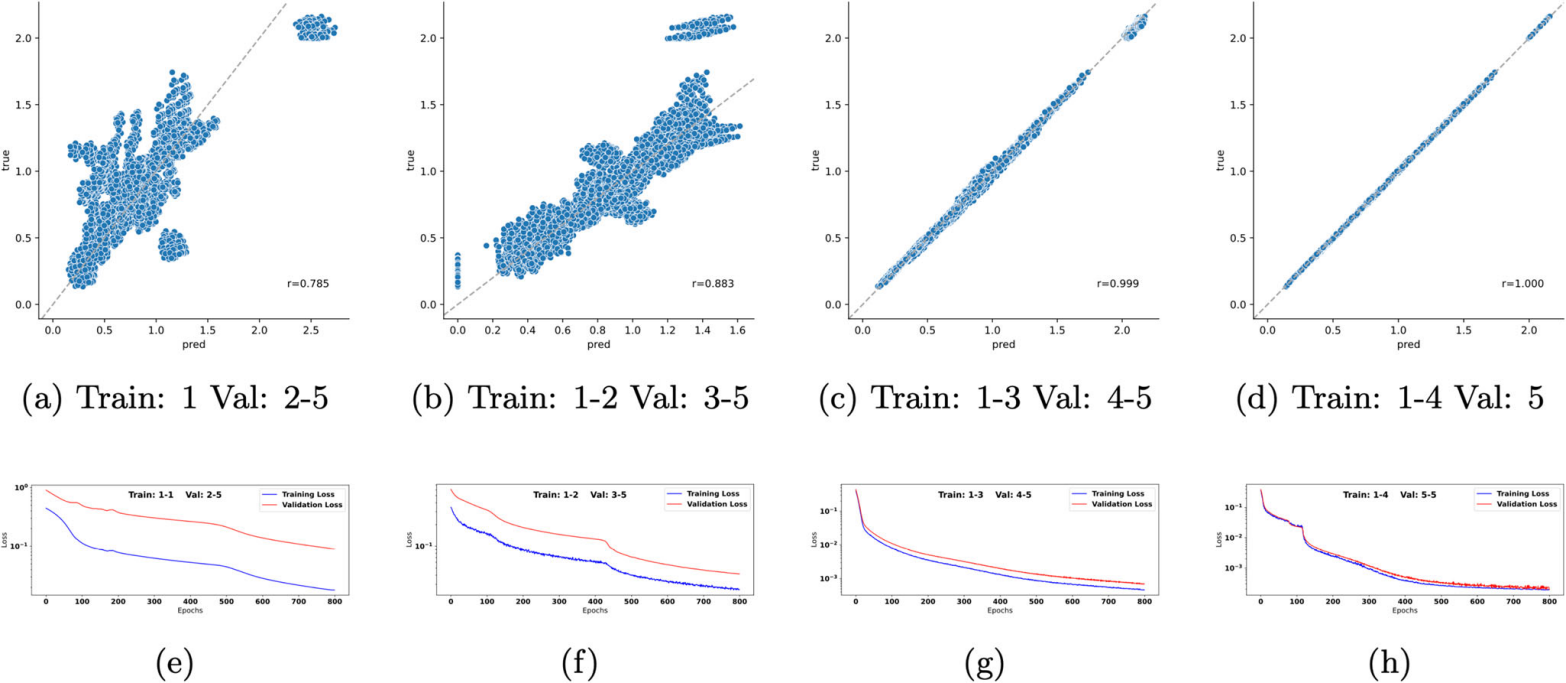
Splitting perturbations by the number of targeted species in the simulation study of paired measurements across multiple targeted perturbations. Here the data was split by single, pairwise and higher order species perturbations. The validation set perturbations always contain combinatorial conditions targeting more species than those used for training. Top row compares the predicted and exact steady states across validation set perturbations for each data split: **a** monospecies perturbations for training and all combinatorial perturbations targeting 2–5 species for validation. **b** Monospecies and pairwise perturbations for training and all combinatorial perturbations targeting 3–5 species for validation. **c**, **d** Are interpreted similarly. The Pearson correlation between predicted and exact values was computed. **e**–**h** Training and validation loss over epochs, corresponding to the splitting above.

To test this scenario, we simulated steady states with *P* chosen to include all monospecies perturbations and all *k*-species combinations for *k* up to 5. We then compared the predicted and exact values of steady states under validation set perturbations, whose size was gradually decreasing as the training set included increasingly more higher order perturbations (Fig. 3a-d). The performance, measured by correlation between predicted and true steady states for all species across all perturbations in the validation set, increased as more information about higher order perturbations were included in the training set. For the most challenging case (Fig. 3a) where only monospecies perturbations were available, we obtained correlation of 0.785. In this case the predicted steady states for some of the higher order perturbations did not match well with the exact values. Adding pairwise species perturbations increased the prediction accuracy. However, once three-way perturbations were added, the predicted and the exact steady states completely aligned and were almost indistinguishable when including four-way perturbations in the training set (Fig. 3c, d). To investigate the relatively large performance jump having included the three-species perturbations (Fig. 3c), we trained MBPert over increasing proportions (25%, 50% and 75%) of 3-species perturbations. Specifically, we kept all one- and two-species perturbations as before, but included subsets of the 120 three-species perturbations. We compared the results between different proportions of three-species perturbations (Supplementary Fig. 2). Our results suggested that whereas including 50% of three-species perturbations might not resolve the discrepancy in prediction for some subset of unseen, higher order perturbations, including a further 25% of three-species perturbations was sufficient for accurate prediction.

When inspecting the loss curves for these cases, (Fig. 3e–h), we found that the gap between training and validation loss narrowed as we moved from the most to the least challenging case. This is not surprising since the training set size increased from just 10 monospecies perturbations to 385 perturbations targeting up to four species, and so it is expected that the model would overfit when the training data size is small.

Yet another simulation design is to train the model using all perturbations in which species *i* are not targeted, and validate on the remaining perturbations targeting species *i*. The motivating question for this design is that if we observe system states without having to perturb species *i*, to what extent can we learn the dynamics and predict system states when *i* is perturbed? We repeated for each species in turn and referred to this process as leave one species out. Of all 1023 perturbation conditions for 10 species, 512 (50%) of them target species *i*, and so in this case the data split for training and validation was roughly equal.

**Fig. 4:**
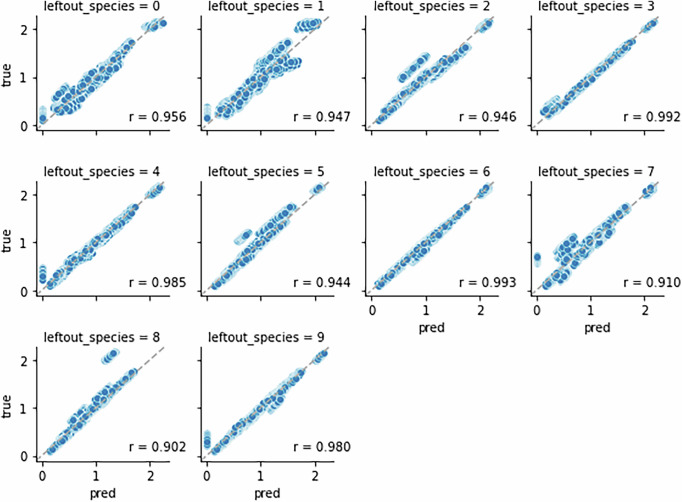
Leave one species out simulation study of paired measurements across multiple targeted perturbations. Here each subfigure shows the predicted versus true steady states under validation set perturbations targeting species *i*, the left-out species. The model was trained using the data of perturbations not targeting the left-out species. Pearson correlations were computed and indicated in each figure.

For each left-out species, the predicted and exact steady states under validation set perturbations aligned well with minimum correlation *r* = 0.902 (Fig. 4). This result suggests that given a new perturbation targeting a previously untargeted species, MBPert is able to predict all species steady states accurately despite not having seen perturbations targeting the species in question during training. A qualitative explanation is as follows. For the simulated data of paired before and after perturbations, the interaction matrix is dense, meaning that the species are coupled, even though the coupling may be weak. This implies that for any species, even though it might not be directly perturbed, its perturbation dynamics can be learned through the perturbation data of other species. In this case, MBPert can effectively estimate species interactions while not requiring complete perturbation data, and can then use the inferred interactions to predict dynamics of all species. Training and validation loss curves corresponding to each left-out species showed convergence of model estimation (Supplementary Fig. 3).

### Simulation study of temporal dynamics with time-dependent perturbations

We again considered 10 species and simulated their trajectory with artificial perturbation introduced at various time points. We compared the estimated and exact gLV parameters and the predicted and observed trajectories for all species.

**Fig. 5:**
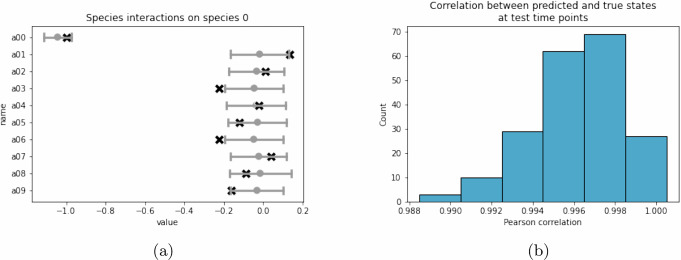
Simulation study for temporal dynamics with time-dependent perturbations, for single group time series data. The trajectories were simulated from 200 random initial states. External perturbations were simulated at different time points. MBPert was trained independently on each time series using time points from the first 120 days and validated on the remaining time points. **a** Interaction effect on species 0. This corresponds to the first row of *A*. As in Fig. 2, the interval was centred on the mean and extended to one standard deviation of the estimate over all simulations. The exact values are indicated by black crosses. The estimated interaction coefficients for species 1–9 were shown in Supplementary Fig. 4. **b** Histogram of correlations between predicted and true states at test time points.

First, time series data for a single group was considered. The majority of gLV parameter estimates contain the exact values within one standard deviation (Fig. 5a, Supplementary Fig. 4, Supplementary Table 1), suggesting that the underlying dynamical system can be recapitulated reasonably well from observations at 24 time points covering 3 perturbation events in a 10 species system.

Using MBPert to predict species dynamics at the 12 future time points, we found excellent agreement between the predicted and true species concentrations at those time points, with the Pearson correlation over 0.99 for most of the simulations (Fig. 5b).

Second, suppose measurements were taken from multiple individuals across different time points, and all individuals underwent the same perturbations throughout the experimental period. If all individuals were frequently sampled then it is possible to train MBPert separately for each individual. However, if some individuals were sparsely sampled, then there would not be sufficient data to accurately infer species dynamics and interactions for those sparsely sampled individuals. To deal with this issue, it is possible to train a single MBPert model by combining data from all individuals, assuming that they share the same underlying dynamics, i.e., we assume the behavior of the species in each group is governed by the same ODE system.

**Fig. 6:**
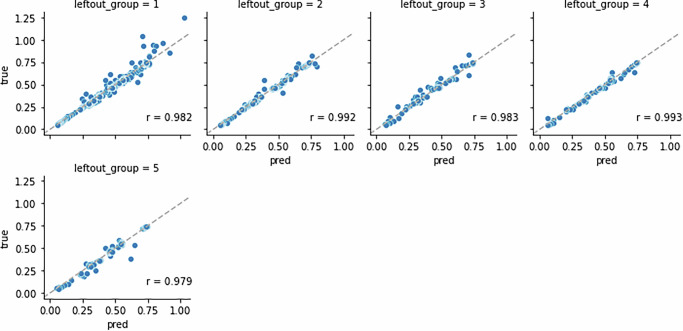
Simulation study for temporal dynamics with time-dependent perturbations, where multiple group time series data was available but some of the groups were sparsely sampled. Leave-one-group-out validation was performed. Each panel plots the estimated and true species concentrations at all time points for the corresponding left-out group. The training and validation loss curves corresponding to each left-out group were shown in Supplementary Fig. 5.

For each left-out group, MBPert was able to predict its dynamics accurately when trained on data of the remaining groups (Fig. 6), achieving excellent Pearson correlation coefficient between the predicted and the exact system states at all time points. In particular, the correlation was still very high (*r* = 0.98) when predicting Group 1 dynamics, for which we had the least amount of training data available compared to predicting the other groups. Training and validation loss curves corresponding to each left-out group showed convergence of model estimation (Supplementary Fig. 5).

### Benchmarking against the MTIST datasets

MTIST (Microbiome Time series Inference Standardized Test simulation)^45^ provides a set of curated datasets to benchmark microbial ecosystem inference algorithms on their ability to infer ecological signs of pairwise species interactions. It consists of 648 realistically simulated microbiome time series with different initial abundances, number of species and individual hosts, varying sampling strategies, sampling frequencies, and amount of noise added to capture unobserved stochastic events. The simulated ground-truth community matrices capture diverse interaction types that are often observed in the human gut microbiome community. For each MTIST dataset, we ran MBPert 50 times and compared the inferred interaction matrix with the ground truth.

**Fig. 7:**
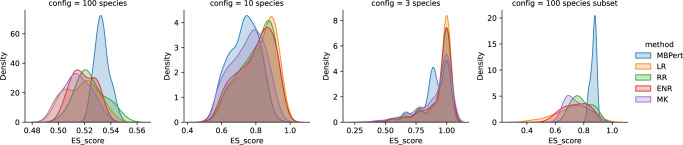
Comparison of the distribution of ES scores, as defined in ref. ^45^, of all interaction type inference methods across 3, 10 and 100-species communities. For 100-species communities, ES scores restricted to only strong-interaction species (∣*a*_*ij*_∣ > 0.25) were also considered. Method abbreviations: LR Linear Regression, RR Ridge Regression, ENR Elastic Net Regression, MK MKSeqSpike.

Although for 3- and 10-species communities MBPert yielded lower average ES scores than other comparison methods, for 100-species communities it was the best performing method, yielding the highest average ES scores both for the case of including all species interactions and restricting to only strong interactions (Fig. 7). Therefore, MBPert was better suited to inferring interaction types for communities with many species, which more closely reflect the scenario encountered in real-world ecological communities. Furthermore, the spread of the ES scores was also much smaller than the other methods, showing that MBPert provided more reliable estimates than other methods. The relatively low ES scores observed in the 3- and 10-species case could be explained by noting that the comparator methods constrained the growth rates such that the equilibrium abundance fell at *x* = 1.0 for each species, whereas MBPert estimated all parameters without such constraint. In contrast, for the 100-species case, the growth rates were instead drawn from a half-normal distribution without constraints on equilibrium abundances^45^, facilitating a fairer comparison between MBPert and the comparator methods.

### Inferring microbial interactions for *Clostridium difficile* infected mouse

To test MBPert on real microbial dynamics data, we used published bacterial time series data of gnotobiotic mouse infection with *Clostridium difficile*^30^. After colonizing with a common set of commensal strains and allowing them to establish the gut microbiota for 28 days, 5 mice were infected with *C. difficile* and monitored for a further 28 days. A total of 26 fecal samples were collected per mouse and subject to 16S rRNA sequencing as well as qPCR to estimate the total biomass, allowing absolute species concentrations to be quantified rather than relative abundances. MBPert was run in a leave-one-mouse-out fashion. The training was converged and the average Pearson correlation between predicted and true species concentrations across all folds was 0.81 (Supplementary Fig. 6).

**Fig. 8:**
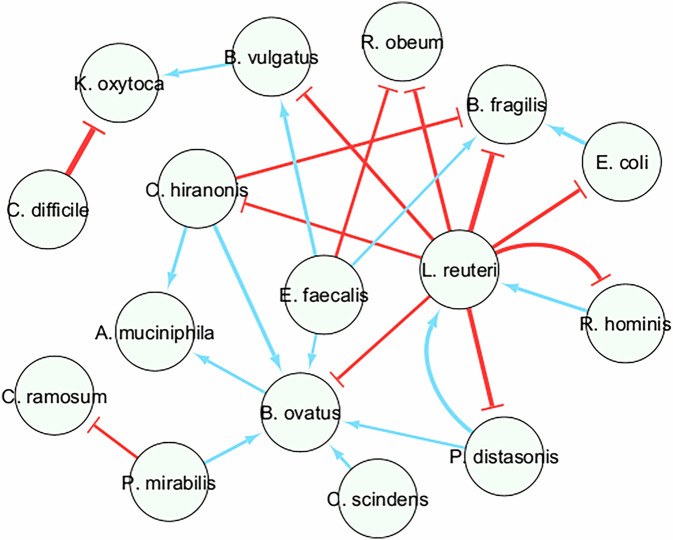
Microbial interaction network for *C. difficile* infected mouse data inferred by MBPert. The sign of the inferred interaction coefficient determines whether it is promotion (positive, blue) or inhibition (negative, red).

Although the inferred interaction network (Fig. 8) did not in general agree with Bucci et al. (Fig. 3a in ref. ^30^), we have found several interesting observations. While both *C. difficile* and *K. oxytoca* are opportinistic pathogens, we have found a strong inhibitory effect exerted by *C. difficile* on *K. oxytoca*, suggesting that *C. difficile* outcompetes *K. oxytoca* for resources in the altered microbiome^46^. *B. ovatus* is a key commensal bacterium in the human gut, particularly involved in the fermentation of complex carbohydrates and dietary fibers, and in the production of short-chain fatty acids (SCFAs). SCFAs produced by *B. ovatus* can indirectly benefit *A. muciniphila* by maintaining intestinal epithelial health and promoting mucus production^47^. We have found *B. ovatus* to be promoted by *C. scindens* and *C. hiranonis*, both of which are part of the *Clostridia* class and involved in carbohydrates fermentation and SCFA production^48^. Another interesting observation is that several species are inhibited by *L. reuteri*, which is a probiotic bacterium with several inhibitory effects on pathogens and harmful bacteria^49^. *L. reuteri* produces reuterin, a broad-spectrum antimicrobial compound derived from fermentation of glycerol and is capable of inhibiting several enteric pathogens such as *Escherichia coli*, *Salmonella*, and *Helicobacter pylori*^50^. It has also been reported that *L. reuteri* inhibits pathogenic adhesion to epithelial cells through competitive exclusion, offering protection of gut epithelia against pathogenic invasions^51^.

### MBPert predicts human gut microbiota dynamics following antibiotic

**Fig. 9:**
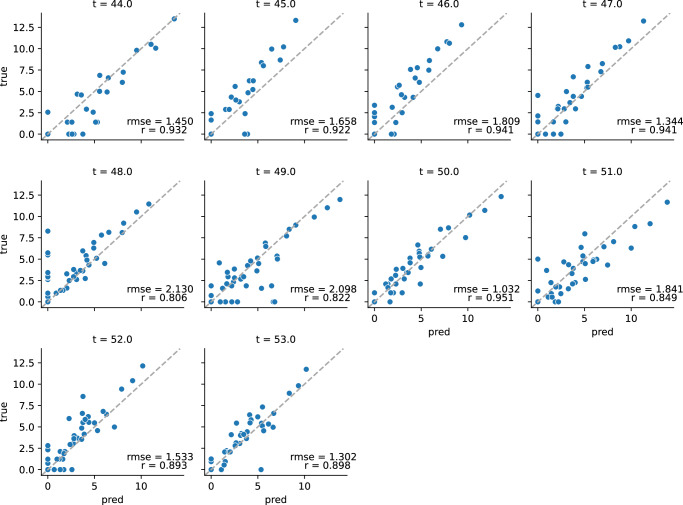
Comparison between predicted and true normalized species abundance across the ten time points after the second antibiotic perturbation. The RMSE and Pearson correlation coefficient were shown in each subplot.

As another example, we seek to use MBPert to predict the future dynamics of the human gut microbiota of one of the patients in the study by^44^. The study examined the impact of repeated antibiotic perturbations on the human distal gut microbiota of three individuals over 10 months, analyzing over 1.7 million bacterial 16S rRNA sequences. Because there were two courses of antibiotics, we trained MBPert on the microbial time series data up to the point where the patient was given a) the first course of antibiotic ciprofloxacin, and b) both courses of the antibiotic, and predicted the dynamics thereafter. We then compared the correlation coefficient as well as the root mean squared error (RMSE) between the predicted and true normalized species abundance across all time points post-antibiotic. When training included both antibiotic perturbation events, the correlation coefficient as well as the RMSE exhibited relatively good performance (Fig. 9), compared to the case where only the first antibiotic perturbation was included (Supplementary Fig. 7), for which there were large RMSE. In that case, the algorithm was not allowed to see the post-antibiotic data after the first ciprofloxacin, and it tended to underestimate the true abundance, with larger RMSE observed immediately after the first antibiotic (*t* = 16) and the second (*t* = 44) (Supplementary Fig. 7). However, judging by the high correlations observed, the trend of species dynamics can be well predicted.

To examine the robustness of the prediction, we trained MBPert with different initializations of the model parameters, and observed that the predicted abundance fell within two standard deviations of the corresponding true value for the majority of species across all time points (Supplementary Fig. 8, Supplementary Table 2). Finally, to estimate the species interaction network, we trained MBPert on all available time points and displayed the inferred species interaction network (Supplementary Fig. 9). A positive relationship between *Bacteroides* and many other species was observed, in agreement with mutualistic roles of *Bacteroides* in the human gut microbiome^52^. Similar symbiotic relationship was observed for *Lachnospiraceae*, members of which also exhibit mutualistic roles with other gut microbial species and the human host^53^.

## Discussion

In this work, we present a generic computational framework for inferring species interactions and dynamics in time-evolving systems that can be perturbed experimentally. Although we have formulated MBPert in the context of microbial dynamics, the underlying framework is readily abstracted to suit a variety of application domains. By using an approximating governing equation–such as the generalized Lotka-Volterra (gLV) equations–to model species interactions, our approach leverages external perturbations, whether targeted or untargeted, to both reveal interaction mechanisms and predict future system states.

MBPert was originally motivated by the challenge of inferring microbial interactions from metagenomic time series data, where species abundances are derived from profiling experiments and are influenced by a range of ecological interactions (e.g., mutualism, commensalism, competition). In microbial systems, perturbations may take the form of targeted bacteriotherapy, untargeted antibiotics, or dietary interventions. Importantly, our method estimates susceptibility values directly from the data without requiring prior knowledge. In addition, the framework’s versatility is underscored by its applicability to other domains–for instance, in the analysis of a drug perturbation experiment in^43^, the system state is defined by protein levels measured via reverse-phase protein arrays and interactions are characterized by protein-protein interactions. There, the perturbations are combinatorial drug perturbations designed to search for optimal drug combinations with therapeutic potential—this is the case of paired measurements from targeted perturbations. In ref. ^43^, a special ODE model was proposed to model protein and cellular responses to drug perturbations. Although the model captures the strength and direction of protein interactions, a theoretical understanding of its stability is lacking. Moreover, the inhibition strengths of different drugs to their targets must be preset and provided as input to the model. This quantity is analogous to the susceptibility values used in our modified gLV equations. Unlike^43^, MBPert estimates them in a data-driven manner. MBpert has been assessed against two primary experimental scenarios. Firstly, combinatorial testing enabling paired before-and-after measurements under different perturbation conditions–from monospecies to higher-order combinations. This controlled setting allows for clear attribution of observed dynamics to specific perturbations. In the second scenario, time-dependent perturbations are integrated with longitudinal sampling, where system states are measured at multiple time points. While these perturbations may not be species-specific, this second approach is often more practical when frequent sampling is logistically or ethically constrained–as exemplified by gnotobiotic mouse experiments with *Clostridium difficile* infection and dietary interventions^30^. In both scenarios, MBPert effectively predicts species dynamics by iteratively optimizing model parameters based on the observed data.

It should be noted that due to the complex interplay between different species in real ecological systems, strongly interacting species may be rare and most species would only interact weakly. Therefore, the interactions inferred by any computational model should be interpreted with caution. In both the *C. difficile* infected mouse data and the human gut microbiome perturbation data, our inferred species interaction networks could be affected by noise. In general, we should avoid over-interpreting such networks when lacking prior knowledge about real interactions. As for method comparison, in additional to simulated data, having data from a controlled, real ecosystem where the ground truth interactions are known would be more convincing.

In the antibiotic perturbation dataset, we only considered the time points where the patient received the antibiotic. However, dose and exposure time can also impact the extent of antibiotic response and therefore species abundance level. In addition, the time period for the disturbed microbial community to reach a relatively stable state post-antibiotic should be assessed in real-data applications. If the timescale is too short, recovery might not have happened; whereas if it is too long, no informative dynamics can be gained. Therefore, setting suitable timescales is important when applying the model to real perturbation data.

Despite its strengths, MBPert has several limitations. Similarly to many existing methods, it requires absolute species abundances rather than relative measures, necessitating accurate estimation of total microbial biomass (e.g., via qPCR), which can be subject to PCR bias^54^. However, as pointed out in ref. ^30^, relative abundance data can still be used as long as the total bacterial biomass remains roughly constant, while in studies involving antibiotic exposures, the effect on biomass is unclear a priori. Here, we demonstrate using the classical perturbation data of^44^ that MBPert can recapitulate the post-perturbation microbial dynamics with only the relative abundance data.

Furthermore, by relying on the gLV equations, MBPert inherently captures only pairwise species interactions, potentially limiting its predictive accuracy in systems where non-linear, higher-order interactions play a significant role. One way to infer the presence of higher-order interactions is by estimating the effective pairwise interactions from community data and compare them to the true pairwise interactions obtained in simplified settings (e.g., monocultures and pairwise communities)^55^. If there are significant discrepancies between true and effective interactions, then this may suggest higher-order interactions. In addition, if the predicted abundances based on pairwise interactions alone deviate markedly from empirical data, this indicates that high-order interactions might be at play. From modeling perspective, including higher-order terms (e.g., a general GLV framework with higher-order interaction terms) can help assess whether they significantly improves model fit to data.

Another limitation is that MBPert cannnot describe metabolic interactions, which are mediated by cross-feeding and metabolic exchange^56^. Although the network edges inferred by MBPert have directionality, indicating potential promotion or inhibition of growth, these interactions are not causal and thus do not provide explicit mechanistic insights.

Future work may address some of these limitations by complementing MBPert with metabolic models, such as stoichiometric metabolic network modeling and consensus genome-scale metabolic reconstructions^57,58^. Incorporating concepts from empirical dynamical modeling (EDM)^59,60^ is another promising avenue. EDM, an equation-free alternative to parametric models, can accommodate non-equilibrium dynamics and non-linearity, potentially leading to better detection of causality and more robust modeling of complex ecological systems.

## Methods

### The general framework

Let ${\bf{x}}(t)=({x}_{1}(t),{x}_{2}(t),\ldots ,{x}_{N}(t))\in {{\mathbb{R}}}^{N}$ denote the system state at time *t*, where each component *x*_*i*_(*t*) is the measurement of species *i* at time *t*. Consider the following two experimental designs.

First, paired measurements across multiple targeted perturbations were available. Let *μ* denote a targeted perturbation regarded as a mapping *μ*: {1, …, *N*} → {0, 1}, such that *μ*(*i*) = 1 if species *i* is targeted by the perturbation and *μ*(*i*) = 0 otherwise. Assume that we observe system states at initial and end time points under a set *S* of perturbations: ${\{{{\bf{x}}}^{\mu }(0)\}}_{\mu \in S}$ and ${\{{{\bf{x}}}^{\mu }(T)\}}_{\mu \in S}$. In general, *T* may be perturbation-dependent as some species may react strongly or not at all to different perturbations, affecting the rate at which the system reaches equilibrium. However, this does not affect the computational framework since in this case the perturbations are administered independently. Hence we write **x**^⋆,*μ*^ ≔ **x**^*μ*^(*T*) for each *μ* in *S*.

Using domain knowledge, we suppose that the species dynamics is described by some ordinary differential equation (ODE) parameterized by *θ*, encompassing all unknown parameters encoding species interactions and perturbation effects,

1 $$\frac{d{{\bf{x}}}^{\mu }(t)}{dt}=f({{\bf{x}}}^{\mu }(t);\theta ).$$

Further suppose that equation (1) can be solved numerically for any given value of *θ* and initial state **x**^*μ*^(0), across all perturbations *μ*.

With this setup, the problem of estimating *θ* can be converted into an optimization problem of minimizing the distance between the predicted and observed system states at time *T*, across all species and all perturbation conditions. Here, the predicted system states are obtained by numerically solving the ODE using our current best estimate of *θ*. To achieve this, we design our loss function of *θ* as comprising a mean-squared error (MSE) term and a regularization term,

2 $$L(\theta )=\sum _{\mu \in S}\mathop{\sum }\limits_{i=1}^{N}{\parallel\!\! {\hat{x}}_{i}^{\mu }(T;\theta )}-{x}_{i}^{\star ,\mu }{\parallel }^{2}+\lambda {\parallel \theta \parallel} ,$$

where ${\hat{x}}_{i}^{\mu }(T;\theta )$ is the numerical solution to (1) with parameter *θ*, integrated over time horizon *T*. The regularization term guards against overfitting, induces sparsity and therefore helps with interpreting the results.

Second, consider longitudinal sampling with possible time-dependent perturbations. Assume that we observe a system at a sequence of time points, (**x**(*t*_1_), **x**(*t*_2_), …, **x**(*t*_*M*_)), where each ${\bf{x}}({t}_{l})\in {{\mathbb{R}}}^{N},\,(l=1,\ldots ,M)$ contains species measurements at time *l*. Between *t*_1_ and *t*_*M*_, the system might be subject to one or more types of time-dependent perturbations, denoted by ${\bf{u}}(t)={[{u}_{1}(t),\ldots ,{u}_{K}(t)]}^{T}$, such that *u*_*j*_(*t*) = 1 if perturbation type *j* is active at time *t* and *u*_*j*_(*t*) = 0 otherwise; *K* is the number of perturbation types.

As before, we model the species dynamics by some ODE parameterized by *θ*,

3 $$\frac{d{\bf{x}}(t)}{dt}=g({\bf{x}}(t),{\bf{u}}(t);\theta ),$$

which can be solved numerically for any given value of *θ*.

We again convert the problem of estimating *θ* into an optimization problem. Here we seek to minimize the MSE between observed and predicted states at successive time points along the trajectory,

4 $$L(\theta )=\mathop{\sum }\limits_{l=1}^{M}\mathop{\sum }\limits_{i=1}^{N}{\parallel\!\! {\hat{x}}_{i,{t}_{l-1}}({t}_{l};\theta )-{x}_{i}({t}_{l}){\parallel }}^{2}+\lambda {\parallel \theta \parallel} .$$

Here, ${\hat{x}}_{i,{t}_{l-1}}({t}_{l};\theta )$ is species *i*’s predicted abundance at time *t*_*l*_, obtained by numerically solving equation (3) using the observed species abundance at *t*_*l*−1_ as the initial values, and the current best estimates for *θ* as the parameters of the ODE. We then iteratively update *θ* to minimize the discrepancy between the observed and estimated values across all time points.

The observed system state **x**(*t*) at time *t* may not be the equilibrium state if there was a perturbation event just before *t*. Conversely, if **x**(*t*) remains roughly constant beyond some *t*_*l*_ and no further perturbation events appear later, then subsequent states **x**(*t*_*l*+1_), **x**(*t*_*l*+2_), … , would match closely with ${\hat{{\bf{x}}}}_{{t}_{l}}({t}_{l+1};\theta )$, ${\hat{{\bf{x}}}}_{{t}_{l+1}}({t}_{l+2};\theta ),\ldots \,$, essentially contribute nothing to the loss function (4) and contain minimum information about the parameters. This observation could guide the experimental design in terms of timing the sampling around perturbation events, as we would like to maximize the information contained in the data about the parameters and minimize unnecessary logistical cost associated with frequent sampling. Notice, however, that unlike BEEM^40^, it is not needed to remove equilibrium data points prior to applying our framework.

### MBPert: learning species dynamics and interaction from microbiome perturbation and time series data

Contextualizing the above generic framework in microbiome dynamical modeling, we propose MBPert, for inferring microbial interactions and dynamics from perturbation and time series data.

In the first case, let us suppose that microbial abundances are profiled at an initial time point from multiple subjects, and that individual differences have no effect on microbial community dynamics. The implication is that the change in abundance level of microbes under any perturbation can be attributed to that perturbation and its downstream effects only, and no other external or host-specific factors contribute to the change in species dynamics. Each subject then undergoes a perturbation targeted at one or more microbes, for example, a targeted bacteriotherapy or any form of antibiotics targeting a class of microbes. The perturbations should be different across subjects, so that we observe potentially different dynamics following the perturbations. At an end time point we again profile species abundances for all subjects, obtaining for each subject a paired measurement of species abundances before and after perturbation.

This description fits into the first scenario in 4.1 (The general framework). A natural choice of the equations of ecological dynamics is the gLV equations. Here, to account for perturbations, we set *f* in equation (1) to be

5 $$f({{\bf{x}}}^{\mu }(t);A,{\bf{r}},{\mathbf{\epsilon }})={\rm{diag}}({{\bf{x}}}^{\mu }(t))({\bf{r}}+A{{\bf{x}}}^{\mu }(t)+{\rm{diag}}({\boldsymbol{\epsilon }}){{\bf{u}}}^{\mu })$$

where ${\dot{x}}_{i}^{\mu }(t)$ is the rate of change of species *i*’s absolute abundance at time *t* under perturbation condition *μ*; *A*, **r**, **ϵ** are the species interaction matrix, vector of growth rates, and vector of overall susceptibility to perturbations for each species, respectively. The vector **u**^*μ*^ is defined as ${u}_{i}^{\mu }=1$ if perturbation *μ* targets species *i*, i.e., *μ*(*i*) = 1, and ${u}_{i}^{\mu }=0$ otherwise. The element *a*_*ij*_ of *A* is the pairwise interaction effect of species *j* on *i*, and *a*_*ii*_ the intra-species effect, which is often assumed to be negative.

For the regularization term, we can set different *λ* for the parameters, *λ*_*A*_∥*A*_*i*≠*j*_∥ + *λ*_**r**_∥**r**∥ + *λ*_**ϵ**_∥**ϵ**∥, where *A*_*i*≠*j*_ denotes the vector formed by the off-diagonal elements of *A*. The *λ*’s reflect regularization balance between different parameters during model training. For large community involving many species, we can set *λ*_*A*_ to be larger than *λ*_**r**_ and *λ*_*ϵ*_, as this would encourage the model to find a sparser solution with respect to *A* and so can help with interpretability. The self-interaction coefficients *a*_*ii*_ are excluded because they need to be negative to ensure community stability. The form of regularization can also be flexible, common choices are L2 or L1, corresponding to ridge-type penalty and lasso-type penalty, respectively. For the integration time span *T*, we may use a rescaled time that is suitable for numerical integration rather than the actual time unit, because large *T* would incur more computational cost as the ODE need to be solved up to *T* in each optimization iteration.

To solve equation (1) for a given *θ*, we used the explicit Runge-Kutta method of order 5(4)^61^, which was the default integration method “RK45” of the solver solve_ivp in Python’s scientific library SciPy^62^. Minimization of the loss function (2) was done by the Adam optimizer^63^.

While experimental setup akin to Case 1 might not be common in microbiome research, longitudinal studies of microbiome to elucidate microbial interactions have been frequently adopted^29–31^. Adopting the model specification in^29^ amounts to setting the function *g* in (3) to be

6 $$g({\bf{x}}(t),{\bf{u}}(t);A,{\bf{r}},{\mathcal{E}})={\rm{diag}}({\bf{x}}(t))({\bf{r}}+A{\bf{x}}(t)+{\mathcal{E}}{\bf{u}}(t)),$$

where ${\mathcal{E}}\in {{\mathbb{R}}}^{N\times K}$ is the matrix containing species susceptibility to each kind of perturbation; **u**(*t*) is the perturbation status vector at time *t* as defined before.

Compared to Case 1, here we can have *K* types of perturbations each of which may be scheduled at different time points throughout the course of the experiment, and they need not be targeting specific microbes. For example, if both inoculation with certain pathogens and dietary interventions were used, then we would have *K* = 2. If only the time series data was observed and no perturbations, then we simply remove the term ${\mathcal{E}}{\bf{u}}(t)$ by setting **u**(*t*) = 0 and exclude ${\mathcal{E}}$ from the parameter list that requires gradients.

In the loss function (4), the regularization term was chosen to be the same as Case 1, except that Frobenius norm was used for ${\mathcal{E}}$. Similarly, we may need to rescale the observed time unit to a realistic time unit for numerical integration. This was done by setting the integration time to be *t* = *t*_*end*_*T*_*l*_/*T*, where *T* = *T*_*M*_ is the final time point in actual unit (e.g. days) and *t*_*end*_ is the end point of numerical integration. We used the same ODE solver and optimizer as Case 1.

### Simulation study

In the case of paired measurements before and after perturbations, for the 10 species setup, we initialized ${a}_{ij} \sim \frac{1}{2\sqrt{10}}{\mathcal{N}}(0,1)$ for *i* ≠ *j*, *a*_*ii*_ = −1, **r** ~ Unif(0, 1), ***ϵ*** ~ Unif(−0.2, 1), and computed the gLV steady states from (1) and (5). Initial state vector was drawn from a log-normal distribution, $\log {{\bf{x}}}^{\mu }(0) \sim \sqrt{0.2}{\mathcal{N}}({\bf{0}},I)$, and replicated across all perturbations *μ*. Regularization strength was set as *λ*_*A*_ = 10^−4^, *λ*_**r**_ = *λ*_*ϵ*_ = 10^−5^. We considered three simulation scenarios that differed in how the perturbation conditions were partitioned during training and validation.

In random splitting, a random subsample of 323 perturbations that targeted all single-species and 50% of each of the *k*-species combinations for *k* = 2, …, 5 was chosen. These perturbations were then split randomly with 70% for training and 30% for validation. This was repeated 200 times.

In splitting by number of targeted species, steady states were simulated under all monospecies and all *k*-species combinations for *k* up to 5. Then, MBPert was trained independently, first on just monospecies perturbations, then on monospecies and pairwise species perturbations. The final model was trained on all perturbations targeting up to four species. In each case, the validation set contained all higher-order perturbations not present in training set. For example, in the first case the validation set contained all perturbations of order 2–5, and in the last case the validation set contained only perturbations of order 5.

In leave one species out, steady states were simulated under all possible combinatorial conditions targeting 10 species. Then, for each speices *i*, MBPert was trained on conditions not targeting *i* and validated on conditions targeting *i*.

In the case of temporal dynamics with time-dependent perturbations, the same initialization of the gLV parameters as before were used. The sampling times span 180 days with varying sampling frequency. One type of perturbation was applied on Day 25, 55, 90 and 125. Species trajectories were simulated using equation (3) with the right-hand side replaced by (6). We considered two scenarios depending upon whether one has access to data from just a single group or from multiple groups. Such groups could be the gut microbiome communities of different individuals. We assumed that all groups share the same microbiota composition but different abundance levels.

For single group time series data, sampling interval was 5 days, and 24 data points corresponding to the first 120 days was used for training, and the remaining data points for validation. The prediction task therefore included predicting the dynamics after the last perturbation event at Day 125, which was not observed during training. Trajectories from different initial species concentrations were simulated to test the robustness of MBPert in estimating the gLV parameters. Specifically, 200 time series were simulated with $\log {\bf{x}}(0) \sim {\mathcal{N}}({\bf{0}},I)$, and MBPert was trained independently on each time series.

For multi-group time series data, species trajectories were simulated from five groups with initial states drawn from a log-normal distribution, $\log {\bf{x}}(0) \sim {\mathcal{N}}({\bf{0}},I)$. One group had full sampling frequency in the 180 day period with samples taken every 5 days, one group had half sampling frequency (every 10 days), and the other three groups had minimal sampling frequency (every 20 days). As a result, only half as many observations are available to Group 2 compared with Group 1, and Group 3-5 had four times less data than Group 1. Leave-one-group-out validation was performed by first training the model using all but the first group, validating on Group 1, and then repeated for each group in turn.

### MTIST benchmarking

MTIST datasets^45^ comprise a collection of simulated datasets resembling major ecological interactions observed in real human gut microbiome. MTIST datasets can be used to test new microbial dynamics inference algorithms, much like the standardized image datasets that have been frequently used to benchmark computer vision algorithms. In addition to synthetic microbiome time series data, MTIST also provides an evaluating metric, “ecological sign” (ES) score, that can be used to compare the performance of different algorithms in estimating the interaction types, including mutualism (+/+), competition (−/−), exploitation (+/−), commensalism (+/0), and amensalism (−/0). Notably, the ES score considers only the signs of pairwise interactions. It is defined as the difference between the number of correct and incorrect signs, scaled to vary between 0 and 1, with a score of one indicating all signs are correctly inferred, and a score of zero if all signs are incorrect. Here, we tested MBPert on all 648 MTIST datasets and compared its performance with other inference algorithms reported in^45^, including linear regression (LR), ridge regression (RR), elastic net regression (ENR), and MKSeqSpike (MK)^64^. Since no perturbation events were present, we removed the term involving **u**(*t*) in (6). We set the regularization strength *λ*_*A*_ = 10^−4^ and *λ*_*r*_ = 10^−5^. A maximum of 600 epochs was used and training stops early if the reduction in loss in any 15-epoch window is less than 0.005. The AMSGrad variant of the Adam optimizer was used with learning rate 0.001.

### *Clostridium difficile* mouse infection

Details of the experimental protocol for the gnotobiotic mouse infection study were described in^30^. Processed data including counts, biomass and metadata were downloaded from 10.5281/zenodo.50624 and transformed into MBPert input. Species with zero abundance level in over 80% of samples across all mice were further removed. Species absolute abundance was obtained by scaling the relative abundance by the average total biomass of the three qPCR replicates. MBPert was run in a leave-one-group-out fashion where one of the mice was left out for validation and the remaining used for training. The final estimates of the interaction coefficients were obtained by averaging over the cross-validated folds. These interaction coefficients formed the edge weights of the interaction network, and only those with absolute weights greater than 0.25 were shown. MBPert was run for 100 epochs using the AdamW optimizer with learning rate 0.001 and weight decay 0.01.

### Antibiotic perturbation of human gut micriobiota

The original study of the human distal microbiota response to repeated antibiotic perturbations can be found in^44^. The data corresponding to Patient F, included in the R package LTNLDA^65^, was obtained by the command data("ps",package = "LTNLDA"). We did CSS normalization on the original count data using the metagenomeSeq package^66^. The data was then split into training and validation sets, and 100 MBPert runs with different parameter initializations were performed. Two splitting points were considered, corresponding to the first and second time points at which the respective course of antibiotic perturbation was completed (*t* = 16 and *t* = 44). To compare the predicted with the exact species abundance at the validation time points, we averaged the prediction over all MBPert runs. To estimate species interactions, we trained MBPert on all time points including both perturbation events, and averaged the estimated matrix *A* over all runs. We kept all edges with ∣*a*_*ij*_∣ > 0.1 in the species interaction network.

## Supplementary information

Supplementary Information

## Data Availability

The simulated data were available at https://github.com/yuanwxu/mbpert/tree/main/data. The MTIST datasets can be found at https://github.com/jsevo/mtist. The *C. difficile* mouse infection data were downloaded from 10.5281/zenodo.50624. The antibiotic perturbation data of human gut microbiome was accessed through the R package LTNLDA.

## Code availability

MBPert is available on https://github.com/yuanwxu/mbpert, along with code to reproduce the results in this paper.
