# Supplementary material for: A computational framework for inferring species dynamics and interactions with applications in microbiota ecology
Source: NPJ Syst Biol Appl. 2025 Aug 5;11:87. doi: 10.1038/s41540-025-00568-0 (PMC12325733; doi:10.1038/s41540-025-00568-0)
Supplement: Supplementary file 1 — Supplementary Information [file 41540_2025_568_MOESM1_ESM.pdf]

Supplementary Data
A computational framework for inferring species dynamics and interactions with applications in microbiota ecology

Yuanwei Xu, Georgios V. Gkoutos

Supplementary Note

Parameter initialization and settings of MBPert

In order to ensure stability of steady state solutions, the interaction matrix A for n species was initialized as

$$A_{ij} \sim \frac{1}{2\sqrt{n}}\mathcal{N}(0,1) \text{ if } i \neq j, \text{ and } A_{ii} = -1,$$

following a theorem stated in [1] (Theorem 17, Supplementary Text), which states that if

$$A_{ij} \sim \frac{1}{\sqrt{(2+\delta)n}}\mathcal{N}(0,1), \quad i \neq j$$

for any $\delta > 0$ and $A_{ii} = -1$, then the random matrix $A \in \mathbb{R}^{n\times n}$ is asymptotically almost surely diagonally stable. The diagonal stability of A guarantees the stability of the gLV equations for all initial states $\mathbf{x}(0)$ in the positive quadrant [2]. Furthermore, this stability is persistent under sufficiently small changes to the parameters (Theorem 3.6.4, [3]), implying that local update to the parameters in MBPert does not alter stability.

For the simulation study of leave-one-species-out in the case of paired measurements across multiple targeted perturbations, we used the AMSGrad variant of the Adam optimizer [4]. In other cases the default settings of Adam in PyTorch was used. In practice, we found that using AMSGrad could improve on numerical instability and divergence associated with Adam, however, with the default learning rate the convergence can be slow. We have added stability check in our ODE solver, which will alert the users if the adaptive step size becomes very small. In practice, we recommend first trying the default Adam optimizer while monitoring its convergence, if an issue was encountered, which based on our experience is often signified by a drastic increase of the loss function, then we recommend lowering the learning rate and/or using the AMSGrad variant to ensure training can proceed without running into numerical problems.

Supplementary Figures

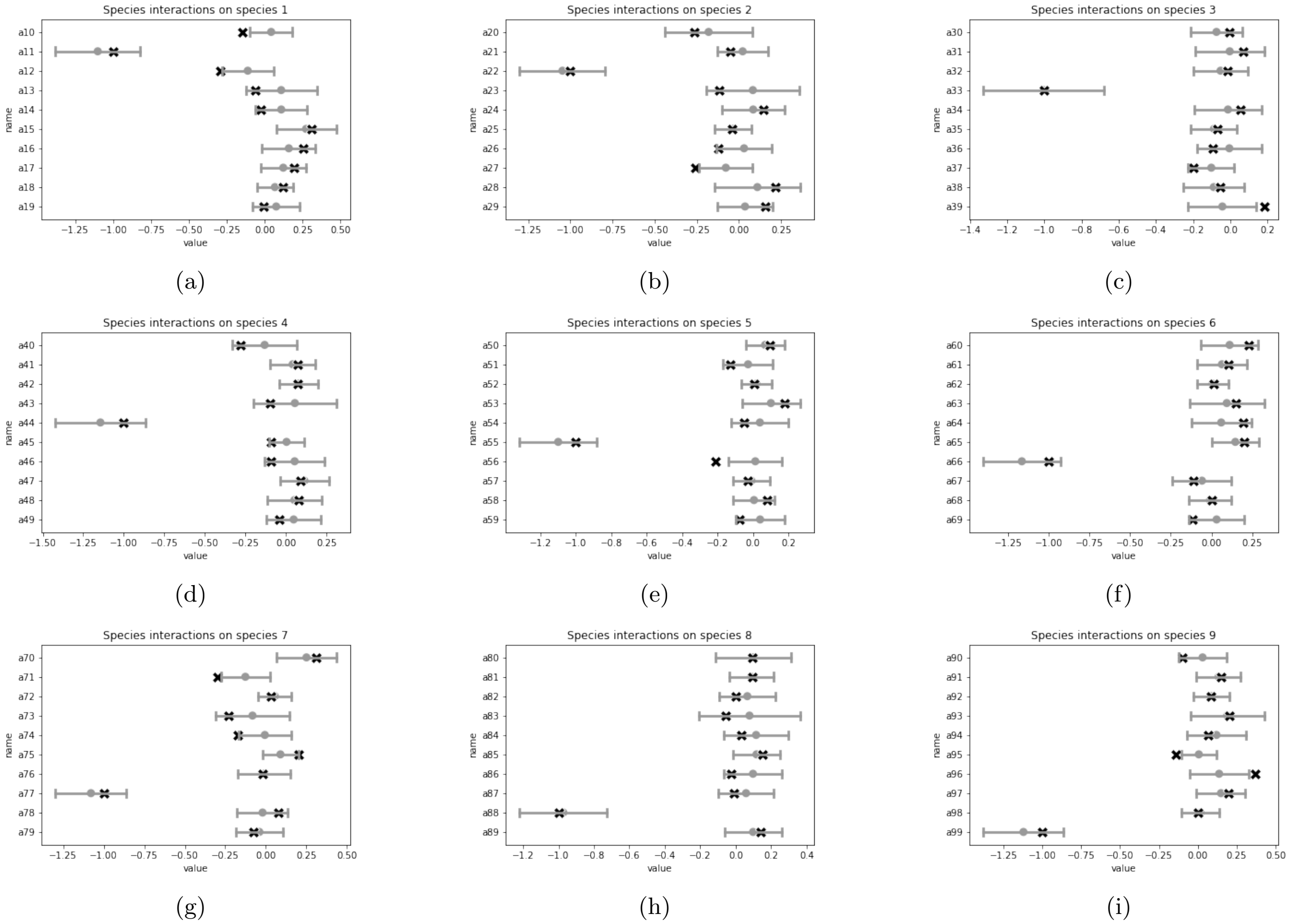

Supplementary Figure 1: Comparison of the estimated interaction effects on species 1-9 with the true values of corresponding rows of A, for the simulation study of pired measurements across multiple targeted perturbations. A random subsample of perturbations targeting up to 5 species were chosen and split into training and validation sets. MBPert was trained on the initial and steady states corresponding to training set perturbations and then evaluated on validation set perturbations. This process was repeated for 200 times. The interval represents the mean and one standard deviation of the MBPert estimate over all random splits. The exact values are indicated by black crosses.

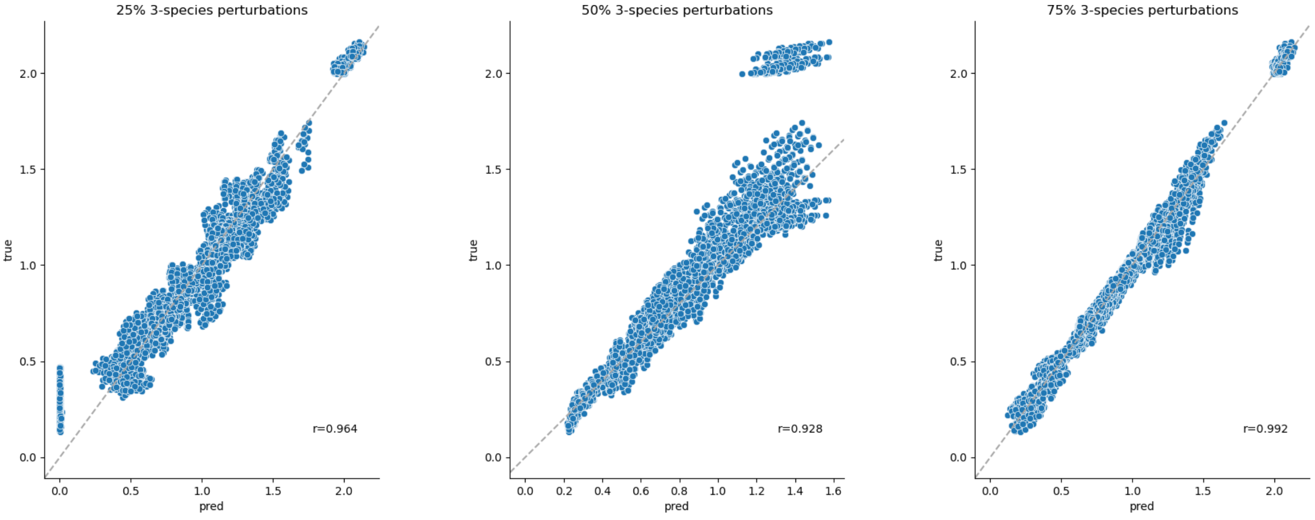

(a) (b) (c)

Supplementary Figure 2: Comparison of predicted and true steady states corresponding to unseen, three-species and higher order perturbations, using simulated data of paired perturbations. The title of each plot indicates the percentage of three-species perturbations added to the training set, which included all one- and two-species perturbations.

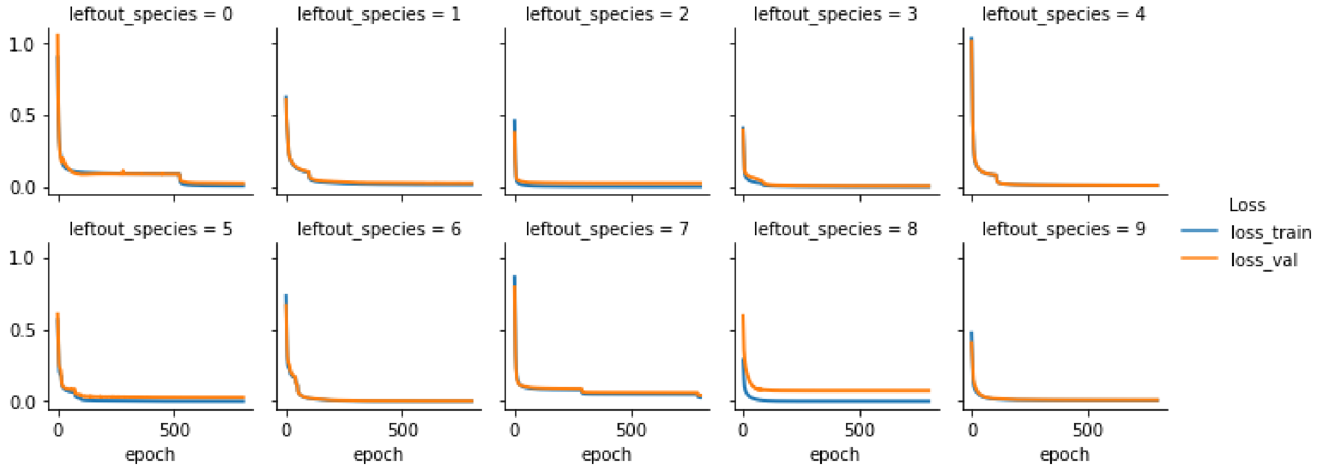

Supplementary Figure 3: Training and validation loss curves for the leave-one-species-out simulation study of paired measurements across multiple targeted perturbations. Each panel shows the corresponding training and validation loss for the left-out species.

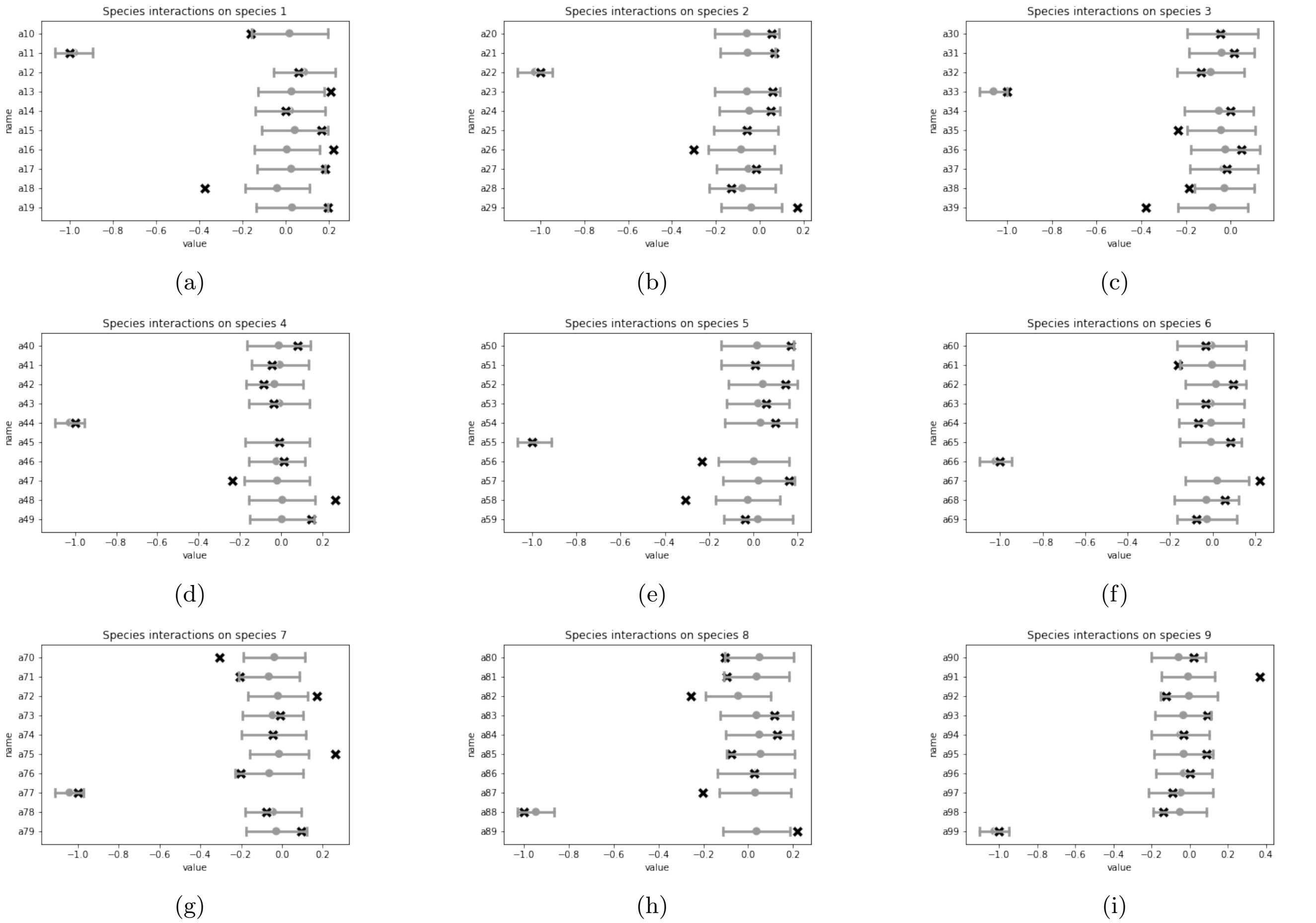

Supplementary Figure 4: Comparison of the estimated interaction effects on species 1-9 with the true values of corresponding rows of A, for the simulation study of single group time series data with time-dependent perturbations. The trajectories were simulated from 200 random initial states. External perturbations were simulated at different time points. MBPert was trained on data points from the first 120 days and validated on the remaining time points. The interval represents the mean and one standard deviation of the MBPert estimate over all simulations. The exact values are indicated by black crosses.

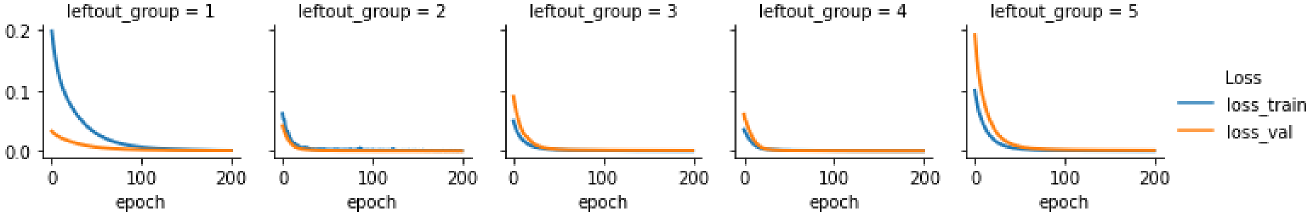

Supplementary Figure 5: Training and validation loss curves for the simulation study of time series data for multiple groups. Leave-one-group-out validation was performed using MBPert. Each panel shows the corresponding training and validation loss for the left-out group.

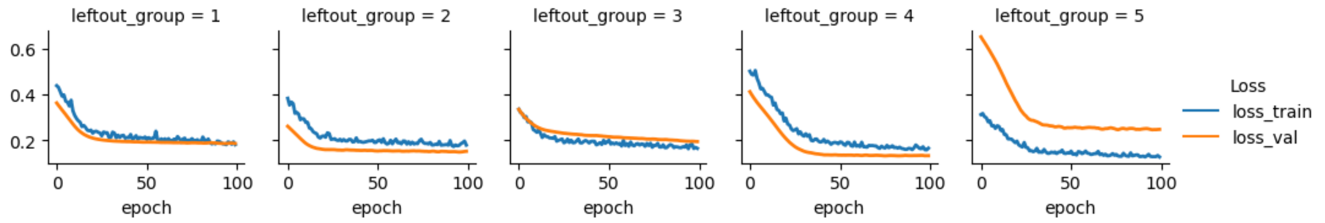

(a)

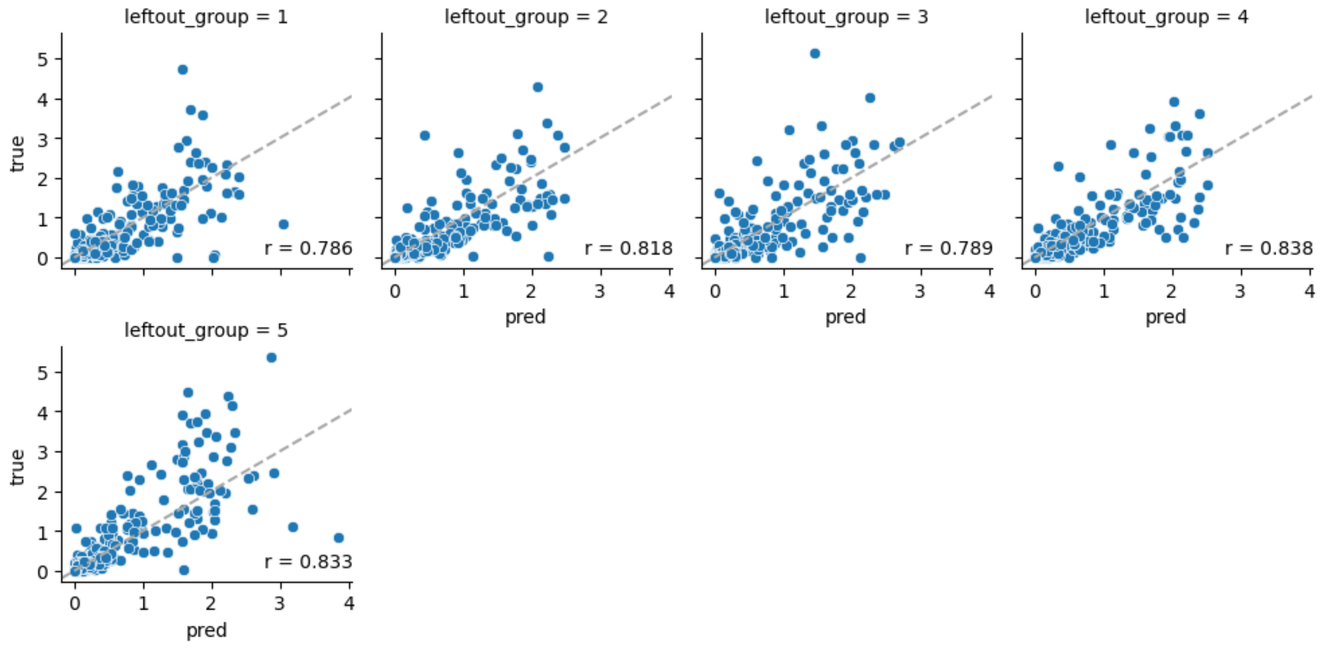

(b)

Supplementary Figure 6: (a) Loss curves of applying MBPert to *C. difficile* infected mouse data; and (b) error between the estimated and true species concentrations at all time points for the left-out mouse, with Pearson correlation coefficient (r) annotated.

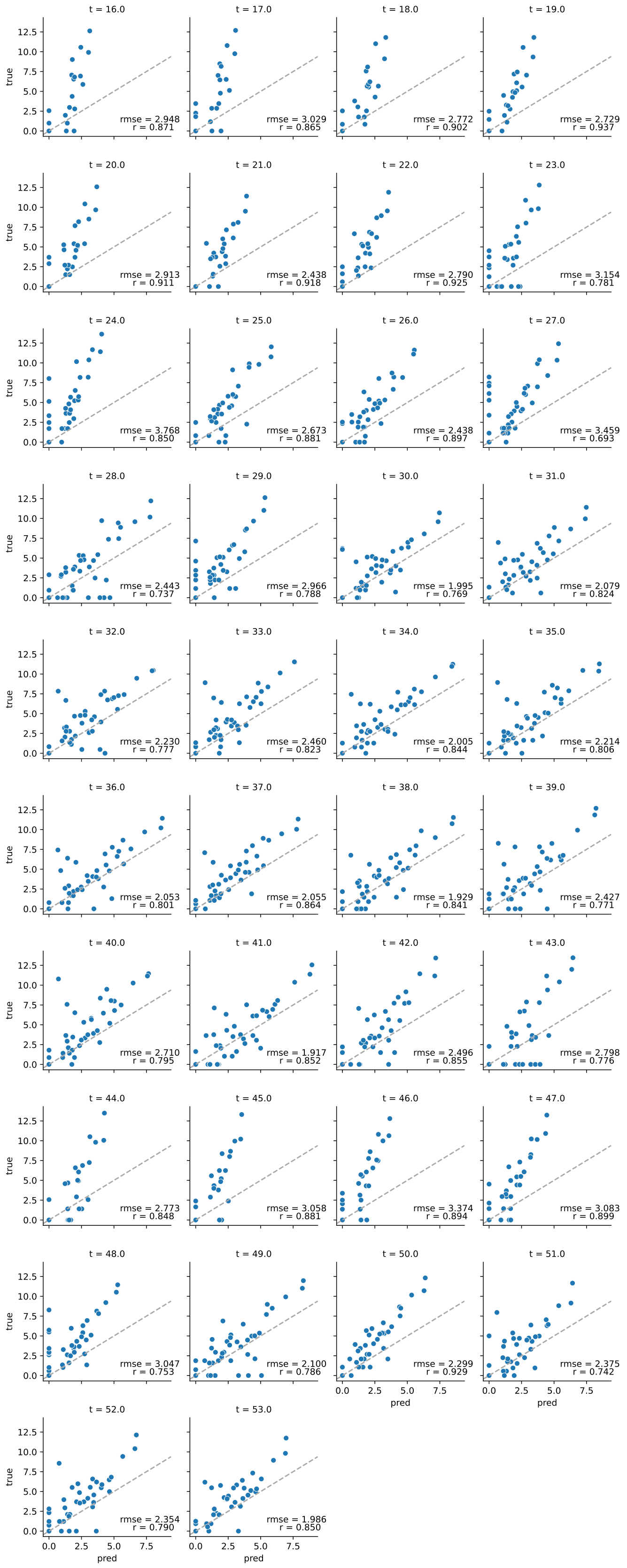

Supplementary Figure 7: Comparison between predicted and true normalized species abundance across all time points ($t = 16 \ldots 53$) after the first antibiotic perturbation. The RMSE and Pearson correlation coefficient were shown in each subplot.

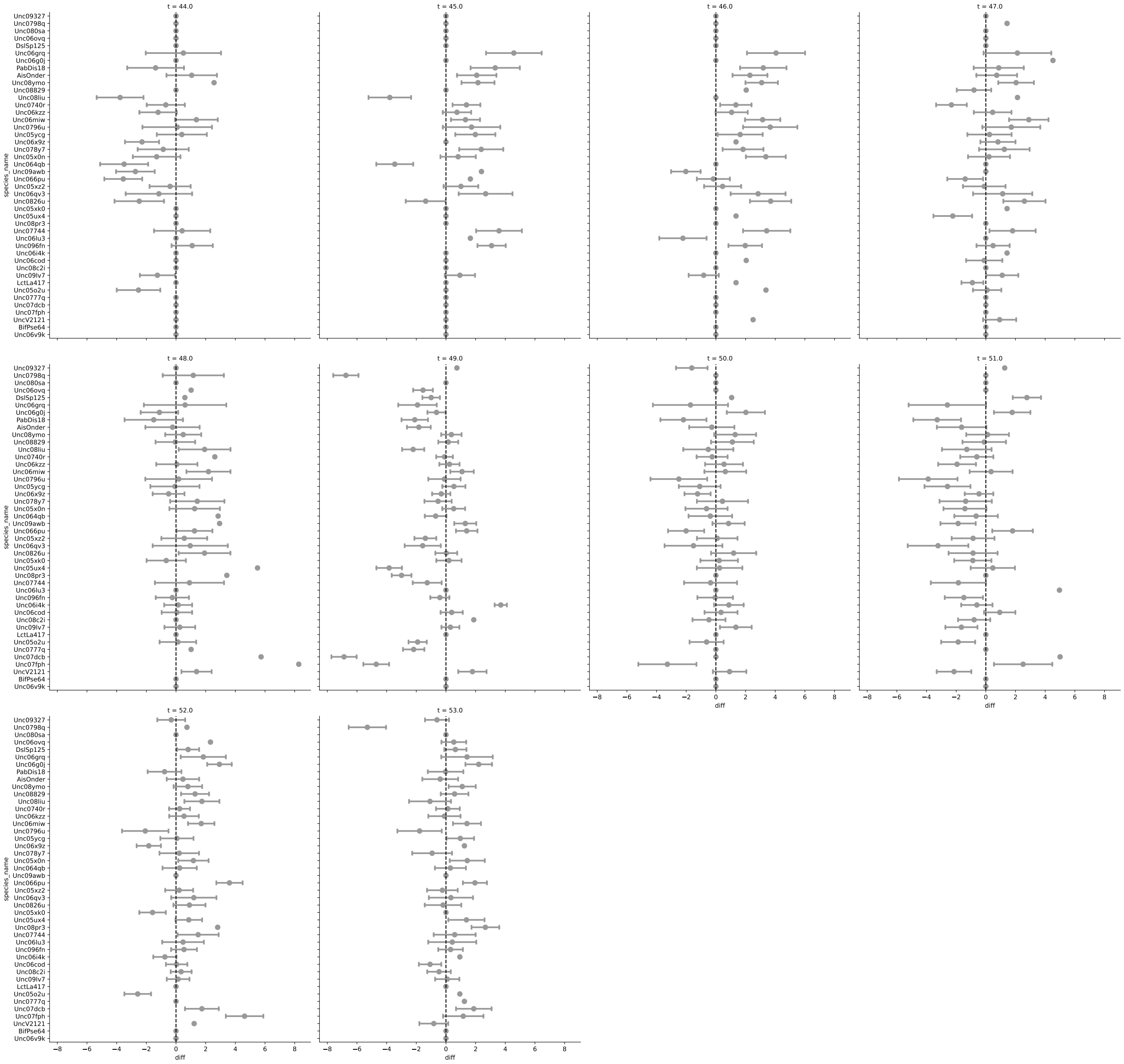

Supplementary Figure 8: For each test time point after the second antibiotic perturbation ($t = 44 \dots 53$), we plot the standard error of the difference between the predicted and true normalized species abundance over 100 MBPert runs with different parameter initializations. The mean was shown in the error bar and it extends to two standard deviations on either side.

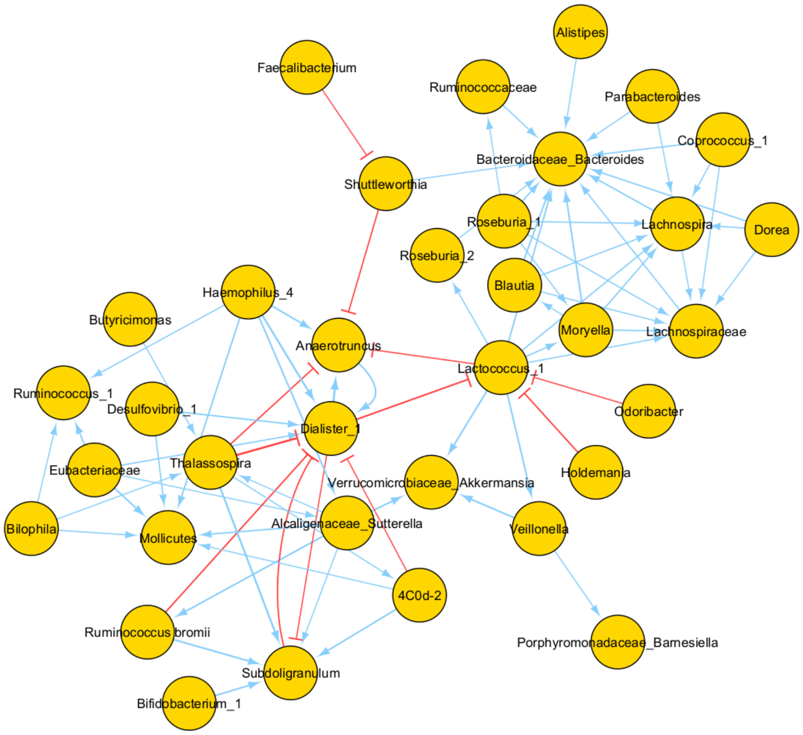

Supplementary Figure 9: MBPert estimated species interaction network for the antibiotic perturbation data of [5]. The sign of the inferred interaction coefficient determines whether it is promotion (positive, blue) or inhibition (negative, red).

Supplementary Tables

Parameter	Within 1 sd	Within 2 sd
A	73/100	98/100
$\mathbf{r}$	8/10	10/10
$\mathcal{E}$	7/10	10/10

Supplementary Table 1: Coverage of true GLV parameters by the corresponding MBPert estimates across 200 simulations with different initial states, for single group, 10 species time series data. For interaction matrix A, 73 out of 100 pairwise interaction coefficients were contained in one standard deviation of the corresponding MBPert estimates, and 98 out of 100 coefficients were contained within two standard deviation. Similarly for the growth rate vector $\mathbf{r}$, 8 out of 10 growth rates were contained in one standard deviation; for susceptibility vector $\mathcal{E}$, the 1-sd coverage was 7 out of 10. Both $\mathbf{r}$ and $\mathcal{E}$ fall within two standard deviation of the MBPert estimates.

	Domain	Phylum	Taxon_3	Taxon_4	Taxon_5	Taxon_6	Taxon_7	Taxon_8
Unc09327	Bacteria	Proteobacteria	Gammaproteobacteria_1	Enterobacteriales_Enterobacteriaceae	Pasteurellales_Pasteurellaceae	Haemophilus_4	Haemophilus_4	Haemophilus_4
Unc0798q	Bacteria	Proteobacteria	Betaproteobacteria	Burkholderiales	Alcaligenaceae_Sutterella	Alcaligenaceae_Sutterella	Alcaligenaceae_Sutterella	Alcaligenaceae_Sutterella
Unc080sa	Bacteria	Proteobacteria	Alphaproteobacteria	Rhodospirillales_1	Rhodospirillaceae	Thalassospira	Thalassospira	Thalassospira
Unc06ovq	Bacteria	Proteobacteria	Deltaproteobacteria	Desulfovibrionales	Desulfovibrionaceae	Bilophila	Bilophila	Bilophila
DslSp125	Bacteria	Proteobacteria	Deltaproteobacteria	Desulfovibrionales	Desulfovibrionaceae	Desulfovibrio_1	Desulfovibrio_1	Desulfovibrio_1
Unc06grq	Bacteria	Bacteroidetes	Bacteroidia_Bacteroidales	Bacteroidaceae_Bacteroides	Bacteroidaceae_Bacteroides	Bacteroidaceae_Bacteroides	Bacteroidaceae_Bacteroides	Bacteroidaceae_Bacteroides
Unc06g0j	Bacteria	Bacteroidetes	Bacteroidia_Bacteroidales	Porphyromonadaceae_Barnesiella	Porphyromonadaceae_Barnesiella	Porphyromonadaceae_Barnesiella	Porphyromonadaceae_Barnesiella	Porphyromonadaceae_Barnesiella
PabDis18	Bacteria	Bacteroidetes	Bacteroidia_Bacteroidales	Porphyromonadaceae_1	Parabacteroides	Parabacteroides	Parabacteroides	Parabacteroides
AisOnder	Bacteria	Bacteroidetes	Bacteroidia_Bacteroidales	Rikenellaceae	Alistipes	Alistipes	Alistipes	Alistipes
Unc08ymo	Bacteria	Bacteroidetes	Bacteroidia_Bacteroidales	Porphyromonadaceae_2	Odoribacter	Odoribacter	Odoribacter	Odoribacter
Unc08829	Bacteria	Bacteroidetes	Bacteroidia_Bacteroidales	Porphyromonadaceae_2	Butyricimonas	Butyricimonas	Butyricimonas	Butyricimonas
Unc08liu	Bacteria	Verrucomicrobia	Verrucomicrobiae_Verrucomicrobiales	Verrucomicrobiaceae_Akkermansia	Verrucomicrobiaceae_Akkermansia	Verrucomicrobiaceae_Akkermansia	Verrucomicrobiaceae_Akkermansia	Verrucomicrobiaceae_Akkermansia
Unc0740r	Bacteria	Firmicutes	Clostridia_1	Clostridiales	Lachnospiraceae	Howardella	Howardella	Howardella
Unc06kzz	Bacteria	Firmicutes	Clostridia_1	Clostridiales	Lachnospiraceae	Coprococcus_1	Coprococcus_1	Coprococcus_1
Unc06miw	Bacteria	Firmicutes	Clostridia_1	Clostridiales	Lachnospiraceae	Coprococcus_2	Coprococcus_2	Coprococcus_2
Unc0796u	Bacteria	Firmicutes	Clostridia_1	Clostridiales	Lachnospiraceae	Lachnospira	Lachnospira	Lachnospira
Unc05ycg	Bacteria	Firmicutes	Clostridia_1	Clostridiales	Lachnospiraceae	Moryella	Moryella	Moryella
Unc06x9z	Bacteria	Firmicutes	Clostridia_1	Clostridiales	Lachnospiraceae	Marvinbryantia	Marvinbryantia	Marvinbryantia
Unc078y7	Bacteria	Firmicutes	Clostridia_1	Clostridiales	Lachnospiraceae	Blautia	Blautia	Blautia
Unc05x0n	Bacteria	Firmicutes	Clostridia_1	Clostridiales	Lachnospiraceae	Roseburia_1	Roseburia_1	Roseburia_1
Unc064qb	Bacteria	Firmicutes	Clostridia_1	Clostridiales	Lachnospiraceae	Roseburia_2	Roseburia_2	Roseburia_2
Unc09awb	Bacteria	Firmicutes	Clostridia_1	Clostridiales	Lachnospiraceae	Shuttleworthia	Shuttleworthia	Shuttleworthia
Unc066pu	Bacteria	Firmicutes	Clostridia_1	Clostridiales	Lachnospiraceae	Roseburia_4	Roseburia_4	Roseburia_4
Unc05xz2	Bacteria	Firmicutes	Clostridia_1	Clostridiales	Lachnospiraceae	Dorea	Dorea	Dorea
Unc06qv3	Bacteria	Firmicutes	Clostridia_1	Clostridiales	Lachnospiraceae	Lachnospiraceae	Lachnospiraceae	Lachnospiraceae
Unc0826u	Bacteria	Firmicutes	Clostridia_1	Clostridiales	Ruminococcaceae	Faecalibacterium	Faecalibacterium	Faecalibacterium
Unc05xk0	Bacteria	Firmicutes	Clostridia_1	Clostridiales	Ruminococcaceae	Subdoligranulum	Subdoligranulum	Subdoligranulum
Unc05ux4	Bacteria	Firmicutes	Clostridia_1	Clostridiales	Ruminococcaceae	Anaerotruncus	Anaerotruncus	Anaerotruncus
Unc08pr3	Bacteria	Firmicutes	Clostridia_1	Clostridiales	Ruminococcaceae	Ruminococcus_1	Ruminococcus_1	Ruminococcus_1
Unc07744	Bacteria	Firmicutes	Clostridia_1	Clostridiales	Ruminococcaceae	Ruminococcaceae	Ruminococcaceae	Ruminococcaceae
Unc06lu3	Bacteria	Firmicutes	Clostridia_1	Clostridiales	Ruminococcaceae	Ruminococcus_2	Ruminococcus bromii	Ruminococcus bromii
Unc096fn	Bacteria	Firmicutes	Clostridia_1	Clostridiales	Ruminococcaceae	Oscillospira	Oscillospira	Oscillospira
Unc06i4k	Bacteria	Firmicutes	Clostridia_1	Clostridiales	Clostridiaceae_1	Clostridium_1	Clostridium	Clostridium
Unc06cod	Bacteria	Firmicutes	Clostridia_1	Clostridiales	Peptostreptococcaceae_1	Peptostreptococcaceae_1	Peptostreptococcaceae_1	Peptostreptococcaceae_1
Unc08c2i	Bacteria	Firmicutes	Clostridia_1	Clostridiales	Eubacteriaceae	Eubacteriaceae	Eubacteriaceae	Eubacteriaceae
Unc09lv7	Bacteria	Firmicutes	Bacilli	Lactobacillales	Streptococcaceae	Streptococcus	Streptococcus	Streptococcus
LctLa417	Bacteria	Firmicutes	Bacilli	Lactobacillales	Streptococcaceae	Lactococcus_1	Lactococcus_1	Lactococcus_1
Unc05o2u	Bacteria	Firmicutes	Erysipelotrichi_Erysipelotrichales	Erysipelotrichi_Erysipelotrichales	Erysipelotrichi_Erysipelotrichales	Erysipelotrichi_Erysipelotrichales	Erysipelotrichi_Erysipelotrichales	Erysipelotrichi_Erysipelotrichales
Unc0777q	Bacteria	Firmicutes	Erysipelotrichi_Erysipelotrichales	Holdemania	Holdemania	Holdemania	Holdemania	Holdemania
Unc07dcb	Bacteria	Firmicutes	Mollicutes	Mollicutes	Mollicutes	Mollicutes	Mollicutes	Mollicutes
Unc07fph	Bacteria	Firmicutes	Clostridia_2	Clostridiales	Veillonellaceae	Dialister_1	Dialister_1	Dialister_1
UncV2121	Bacteria	Firmicutes	Clostridia_2	Clostridiales	Veillonellaceae	Veillonella	Veillonella	Veillonella
BifPse64	Bacteria	Actinobacteria	Actinobacteridae	Bifidobacteriales_Bifidobacteriaceae	Bifidobacterium_1	Bifidobacterium_1	Bifidobacterium_1	Bifidobacterium_1
Unc06v9k	Bacteria	4C0d-2	4C0d-2	4C0d-2	4C0d-2	4C0d-2	4C0d-2	4C0d-2

Supplementary Table 2: Correspondence between species names in Figure 7 and their taxonomic ranks.

Supplementary References

[1] Gibson, T. E., Bashan, A., Cao, H.-T., Weiss, S. T. & Liu, Y.-Y. On the origins and control of community types in the human microbiome. *PLOS Computational Biology* **12**, 1–21 (2016). URL `https://doi.org/10.1371/journal.pcbi.1004688`.

[2] Goh, B. S. Global stability in many-species systems. *The American Naturalist* **111**, 135–143 (1977). URL `http://www.jstor.org/stable/2459985`.

[3] Wiggins, S. *Introduction to applied nonlinear dynamical systems and chaos*, vol. 2 (Springer, 2003).

[4] Reddi, S. J., Kale, S. & Kumar, S. On the convergence of adam and beyond. *arXiv preprint arXiv:1904.09237* (2019).

[5] Dethlefsen, L. & Relman, D. A. Incomplete recovery and individualized responses of the human distal gut microbiota to repeated antibiotic perturbation. *Proceedings of the National Academy of Sciences* **108**, 4554–4561 (2011). URL `https://www.pnas.org/doi/abs/10.1073/pnas.1000087107`. `https://www.pnas.org/doi/pdf/10.1073/pnas.1000087107`.
